# Long Noncoding RNA FOXP4-AS1 Predicts Unfavourable Prognosis and Regulates Proliferation and Invasion in Hepatocellular Carcinoma

**DOI:** 10.1155/2021/8850656

**Published:** 2021-02-01

**Authors:** Jingchen Liang, Duo Wang, Guanhua Qiu, Xiaoqi Zhu, Junjie Liu, Hang Li, Pingping Guo

**Affiliations:** Department of Ultrasound, Affiliated Tumor Hospital of Guangxi Medical University, Nanning, Guangxi Zhuang Autonomous Region 530021, China

## Abstract

**Background:**

Hepatocellular carcinoma (HCC) is the most common type of primary liver cancer that has a high level of morbidity and mortality. Long noncoding RNA (lncRNA) is a novel regulatory factor of tumour proliferation, apoptosis, and metastasis. Our previous studies indicated that lncRNA FOXP4-AS1 is a functional oncogene in HCC; thus, this study is aimed at further evaluating the clinical and biological function of FOXP4-AS1 in HCC. *Material and Methods*. First, we detected the expression of FOXP4-AS1 in HCC tissues and paracarcinoma normal tissues by qRT-PCR. Second, the prognostic effects of FOXP4-AS1 in patients with HCC were analysed in a training group and a verification group. Subsequently, to investigate the biological effects of FOXP4-AS1 on HCC cells, downexpression tests were further conducted.

**Results:**

The expression of FOXP4-AS1 was higher in HCC tissues than adjacent nontumourous tissues, whereas the low expression of FOXP4-AS1 was correlated with optimistic treatment outcomes, which suggested that FOXP4-AS1 may be an independent prognostic biomarker for HCC. Moreover, the downregulation of FOXP4-AS1 significantly reduced the cell proliferation and clonal abilities and inhibited the invasion, migration, and angiogenesis of hepatoma cells (*P* < 0.05).

**Conclusion:**

These results revealed the clinical significance and biological function of FOXP4-AS1 in HCC development, which may provide a new direction for finding therapeutic targets and potential prognostic biomarkers of HCC.

## 1. Introduction

Hepatocellular carcinoma (HCC) is one of the most common malignant tumours worldwide. Approximately 700,000 new cases are diagnosed every year [1]. Although great progress has been made in the treatment for HCC in recent years, including surgical resection, liver transplantation, radiofrequency ablation, interventional therapy, and drug targeted therapy, the overall prognosis of patients with HCC remains poor owing to the high rates of recurrence and metastasis [2]. Therefore, it is essential to study the pathogenesis of liver cancer and finding tumour markers with high sensitivity and specificity to provide a more reliable basis for the early diagnosis, effective treatment, and prognosis of liver cancer.

Recently, long noncoding RNAs (lncRNAs) have gradually entered the field of vision of researchers. lncRNAs are a type of noncoding RNA with >200 nucleotides; lncRNAs can affect many biological processes such as epigenetic modification, transcription regulation, protein translation, and degradation [3, 4]. More and more new lncRNAs have been found and identified as tumour suppressor genes in human cancer, which provides the possibility that lncRNAs may function as new tumour biomarkers and therapeutic targets [5, 6]. Currently, lncRNA forkhead box P4 antisense RNA 1 (FOXP4-AS1), which is a lncRNA related to tumours, is believed to participate in the occurrence of tumours and promote tumour proliferation, invasion, and migration; thus, its upregulation is usually related to tumour grade and poor prognosis [7, 8]. Nevertheless, the capability of FOXP4-AS1 in the development of HCC remains unclear.

This study is aimed at detecting the FOXP4-AS1 expression level in HCC tissues and at investigating its effect on prognosis. Furthermore, in vitro experiments are aimed at examining the impact of FOXP4-AS1 on the biological function of HCC cells.

## 2. Materials and Methods

### 2.1. Clinical Sample Collection

A total of 121 pairs of surgical specimens, containing tumour tissues and paracarcinoma normal tissues, from patients with liver cancer, were collected from the Affiliated Tumour Hospital of Guangxi Medical University. All patients in the group had no loss of follow-up and complete clinicopathological data. Among them, the training group recruited 87 cases from January 2014 to December 2014, and the validation group enrolled 34 patients from January 2015 to December 2015. All patients underwent radical hepatectomy and were diagnosed as having HCC by pathology and did not receive radiotherapy or chemotherapy before surgery. The adjacent normal liver tissues were collected >2 cm away from the tumour boundary, and the tumour-free liver tissues were confirmed by pathology. The collected tissues were immediately stored in a refrigerator at −80°C. This study was ratified and supervised by the ethics committee of our hospital.

### 2.2. Cell Lines and Transfection

The human hepatoma cell lines Hep3B and Huh7 were preserved in our laboratory. The cells were cultured in Dulbecco's modified Eagle's medium supplemented with 10% foetal bovine serum (Gibco, USA) and grown in a wet incubator at 37°C and 5% CO_2_. With the assistance of Shanghai GeneChem Co., Ltd., the siRNA target sequence design was completed (3E+8 TU/ml). Cell transfection was performed using a transfection reagent (Thermo Scientific, USA) according to the manufacturer's instructions. After 48 hours of transfection, the cells were collected for further study.

The experimental transfection of the siRNA sequence is as follows: FOXP4-AS1-siRNA:5′- TGGCAACCTAGTAACCATTAA-3′; scramble-siRNA: 5′- TTCTCCGAACGTGTCACGT-3′.

### 2.3. qRT-PCR

RNA from tissue samples and cells was extracted using the TRIzol reagent (Thermo Scientific, USA). Subsequently, the qualified RNA was retrotranscribed into cDNA using the PrimaScript™ RT Reagent Kit with gDNA Eraser (Takara Biotechnology, Dalian, China). The RNA expression was detected by RT-PCR using the FastStart Universal SYBR Green Master (Rox) kit (Roche, Germany). The correlation expression level was calculated by the Livak (2 − ΔCT) method with *β*-actin as a reference. The primer sequences of FOXP4-AS1 were 5′-TCGGGTGGAAGTCGTTGC-3′ and 5′- CCTCCGCTTGTCTCCCTTT-3′; the primer sequences of *β*-actin were 5′-TGCGTGACATTAAGGAGAAG-3′ and 5′-GTCAGGCAGCTCGTAGCTCT-3′.

### 2.4. CCK-8 Assay

Cell Counting Kit-8 (CCK-8, Tongren, China) was utilized to evaluate cell proliferation. The cells were inoculated in a 96-well plate at a density of 2 × 10^3^ cells/well and cultured for the indicated times (24, 48, 72, 96, or 120 hours). Afterwards, 10 *μ*l of CCK-8 solution was added into each pore and incubated at 37°C for 100 minutes. The absorbance at 450 nm was measured using a Thermo enzyme reader (Thermo Fisher Scientific, USA).

### 2.5. Colony Formation Assay

A total of 500 hepatoma cells per well were inoculated into 6-well plates. Colony formation after routine culture was conducted for 2 weeks. Cells were washed with phosphate-buffered saline, fixed with methanol, and then stained with 0.1% crystal violet (Sinopharm Chemical Reagent Co., Ltd., China). Visible colonies were counted and imaged by the microscope.

### 2.6. Wound Healing Assay

Hepatoma cells were inoculated into 6-well plates and then incubated overnight. The wound was first scratched with the tip of a 200-*μ*l straw and then cultured in serum-free medium mixed with cytarabine (5 *μ*mol/l). Photos were taken at 0, 24, and 48 hours (≥3 visual fields/hour), and the migration area of each group of photos was calculated with the ImageJ software. Later, the migration area was calculated according to the following formula: migration area (nh) = blank area (0 h) − blank area (nh).

### 2.7. Transwell Assay

The cell density was adjusted to 2 × 10^5^ cells/ml with serum-free medium, then added 100 *μ*l to the upper chamber of the transwell. Meanwhile, 500 *μ*l of complete medium was placed into the lower chamber and then cultured for 48 hours. The cells were routinely fixed, stained, and then imaged using a fluorescence inverted routine microscope (Olympus, Japan) in 5 random 200x microscopic fields. The crystal violet at the bottom of the chamber was eluted with 300 *μ*l of 33% acetic acid. Notably, 100 *μ*l was added into each well of the 96-well plates, and the OD value at 590 nm was read using an enzyme labelling instrument. Different from the migration experiment, the invasion experiment was coated with a layer of Matrigel matrix (50 *μ*l, 1-1.5 mg/ml) (Corning, USA) in the transwell chamber. The rest of the operation is the same as the migration test.

### 2.8. Angiogenesis Assay

Different groups of cells were seeded on a 6-well plate and cultured in serum-free medium for 24 hours after adhering to the wall, then collected the supernatant culture medium. Human umbilical vein endothelial cells (HUVECs) were digested and centrifuged and then suspended with a preestablished supernatant medium and inoculated into a 24-well plate coated with 70 *μ*l Matrigel™ matrix (8-12 mg/ml) (BD, USA) at 1.5 × 10^4^/well and cultured in a carbon dioxide incubator at 37°C for 24 hours. Then, calcein was added to the culture and photographed.

### 2.9. Statistical Analysis

The SPSS 24.0 software (SPSS, Chicago, IL, USA) was used for data analysis, whereas the graphics were created by the GraphPad Prism 8 software (GraphPad Software Inc., La Jolla, CA, USA). *T*-test, *χ*^2^ tests, and multivariate Cox regression analysis were used to assess the significant differences between the data groups. All data were expressed as the mean ± standard of the mean. *P* < 0.05 was considered statistically significant.

## 3. Results

### 3.1. Upregulation of FOXP4-AS1 Expression in Patients with HCC

**Figure 1:**
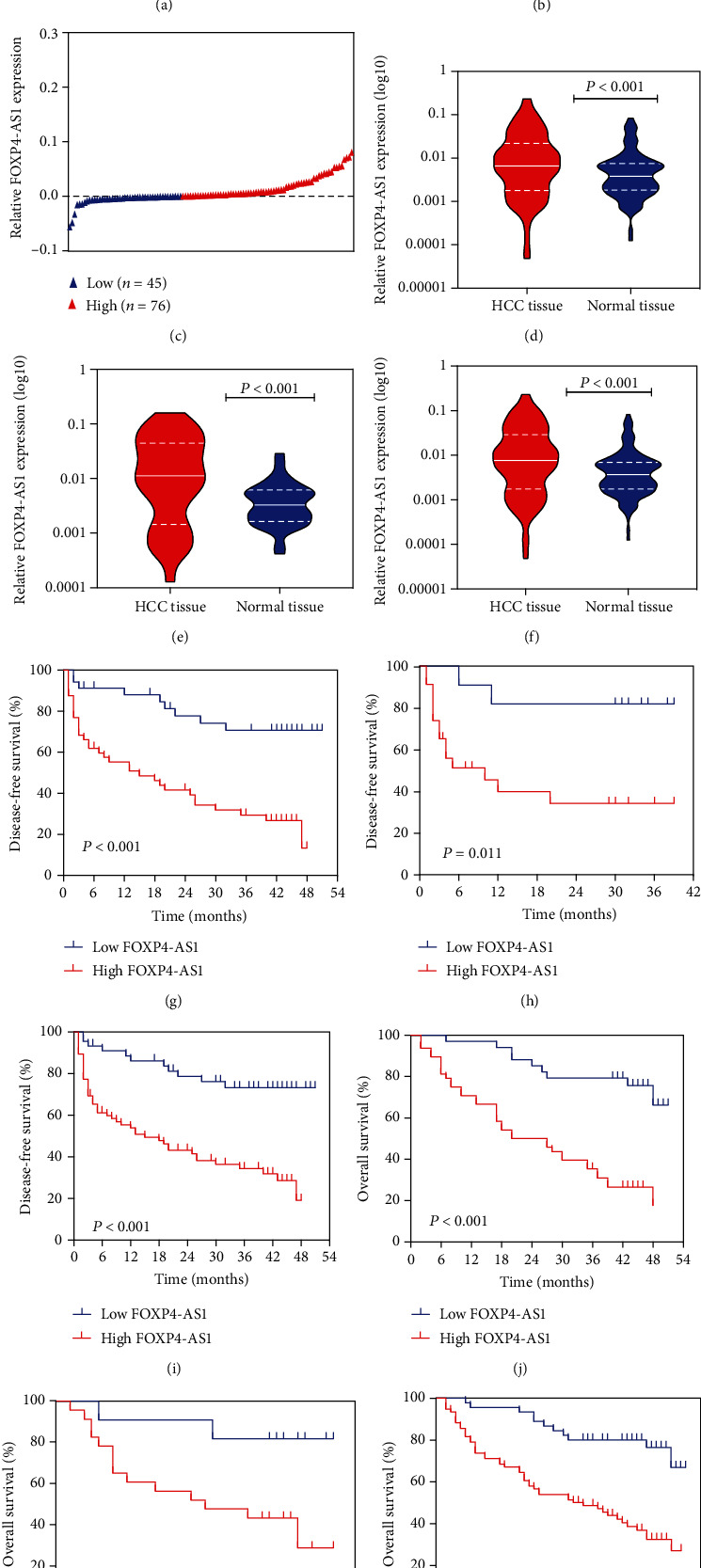
Expression of FOXP4-AS1 in HCC and its correlation with clinical outcome. Patients were divided into groups based on the difference of FOXP4-AS1 expression between HCC and adjacent normal tissues: (a) training set, (b) validation set, and (c) combined set. Compared with adjacent nontumor tissues, the FOXP4-AS1 expression was downregulated in the training set (d), validation set (e), and combination set (f) using the qRT-PCR assay. The DFS and OS of HCC patients with high or low FOXP4-AS1 expression in the training set (*n* = 87; (g), (j), validation set (*n* =34; (h), (k)) and combined set (*n* = 121; (i), (l)) were compared by the Kaplan-Meier survival analysis.

The expression of FOXP4-AS1 was detected in 121 cases of HCC tissues and adjacent normal liver tissues. Meanwhile, the combination set of the training set and validation set was also analysed. We then compared gene expression in HCC tissues and adjacent normal tissues of each patient and divided each group into high- and low-expression groups (Figures 1(a)–1(c)). For the training set, the expression level of FOXP4-AS1 in HCC tissues was considerably upregulated compared with the adjacent nontumour tissues (*P* < 0.001, Figure 1(d)). For the validation dataset, the expression of FOXP4-AS1 was also evidently increased (*P* < 0.001, Figure 1(e)). These are consistent with the results of the combined group (*P* < 0.001, Figure 1(f)).

### 3.2. Correlation between FOXP4-AS1 and Clinical Parameters

We built the relationship between the FOXP4-AS1 expression level and clinical symptoms. In the training group, the FOXP4-AS1 level in HCC samples was associated with age (*P* = 0.001), alpha-fetoprotein (AFP) (*P* = 0.002), and tumour diameter (*P* < 0.001). FOXP4-AS1 level was strikingly related to age, AFP, and tumour diameter (*P* = 0.001, *P* < 0.001, and *P* < 0.001, respectively) in the combined set (Table 1). Subsequently, the disease-free survival (DFS) and overall survival (OS) of different groups were compared according to the FOXP4-AS1 level. Kaplan-Meier analysis revealed that patients with higher FOXP4-AS1 level in the training, verification, and combined sets had shorter DFS (*P* < 0.001, *P* = 0.011, and *P* < 0.001, respectively; Figures 1(g)–1(i)). Similarly, the patients in the 3 groups with higher FOXP4-AS1 revealed poor OS values (*P* = 0.006, *P* = 0.023, and *P* < 0.001, respectively; Figures 1(j)–1(l)).

Besides, in the multivariable Cox regression analysis, in the training group, FOXP4-AS1 was an independent predictor for DFS (hazard ratio (HR), 2.574; 95% confidence interval (CI), 1.126-5.885; *P* = 0.025) and OS (HR, 2.712; 95% CI, 1.140-6.450; *P* = 0.024). In the validation group, FOXP4-AS1 overexpression was an independent predictor for both DFS and OS (HR, 6.826; 95% CI, 1.228-37.945; DFS, *P* = 0.028; HR, 6.505; 95% CI, 1.165-36.399; OS, *P* = 0.033). In the combined set, the FOXP4-AS1 level was an independent predictor for DFS and OS (HR, 2.548; 95% CI, 1.249–5.195; DFS, *P* = 0.010; HR, 3.012; 95% CI, 1.441-6.297; OS, *P* = 0.003) (Table 2). Taken together, a high FOXP4-AS1 expression may be associated with poor prognosis of patients with HCC.

### 3.3. Downregulation of FOXP4-AS1 Inhibited HCC Cell Proliferation, Migration, and Invasion

**Figure 2:**
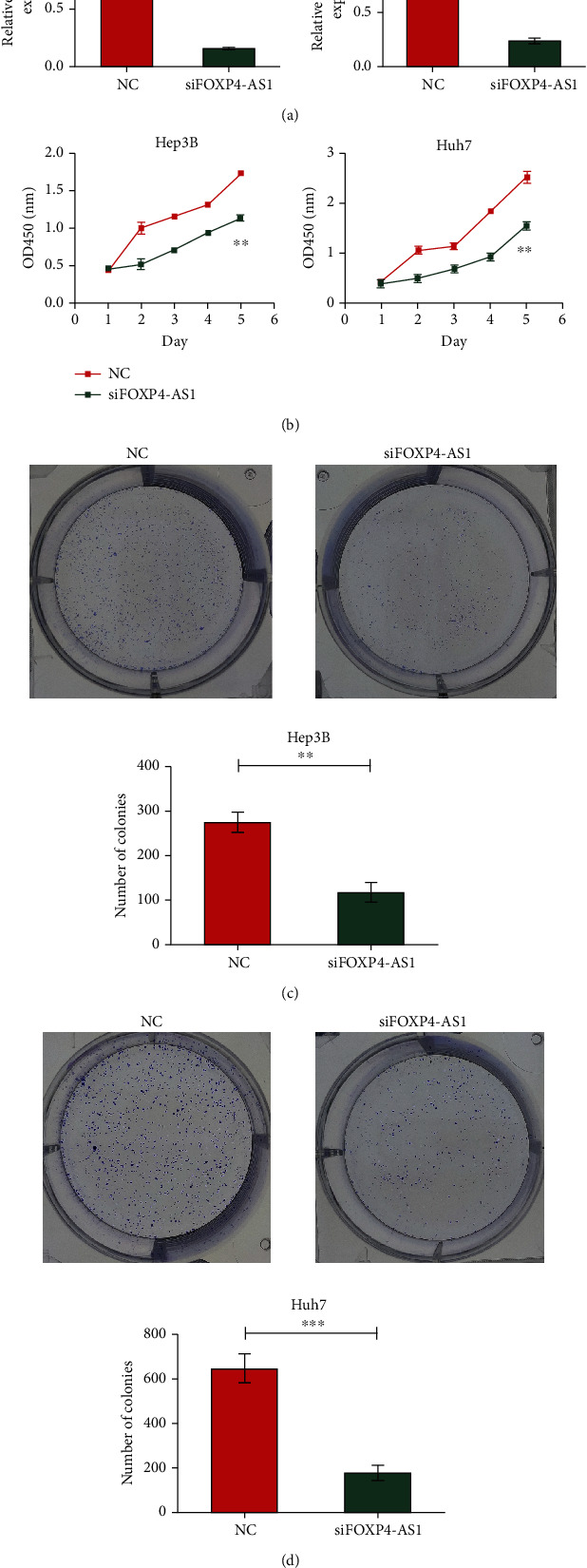
Effect of FOXP4-AS1 on the proliferation of hepatoma cells. (a) Silencing effect of siRNA on FOXP4-AS1 in Hep3B and Huh7 cells. (b–d) The proliferation of Hep3B and Huh7 cells treated with NC or si-FOXP4-AS1 was detected by CCK8 or colony-forming assay. All data are representative of three independent experiments and presented as mean ± s.d.

The silencing effect of siRNA was validated in the Hep3B and Huh7 cell lines. We defined the nonsilenced group as the NC group (control group) and the silencing group as the siFOXP4-AS1 group. The RT-PCR results revealed that the siRNA designed by us had a significant silencing effect on both cell lines (*P* < 0.01, Figure 2(a)). Then, the results of CCK-8 indicated that downregulated FOXP4-AS1 obviously restrained the cell proliferation (*P* < 0.01, Figure 2(b)). Similarly, colony formation assay indicated that knockdown of FOXP4-AS1 inhibited the colony numbers of Hep3B (*P* < 0.01, Figure 2(c)) and Huh7 (*P* < 0.001, Figure 2(d)) cells.

**Figure 3:**
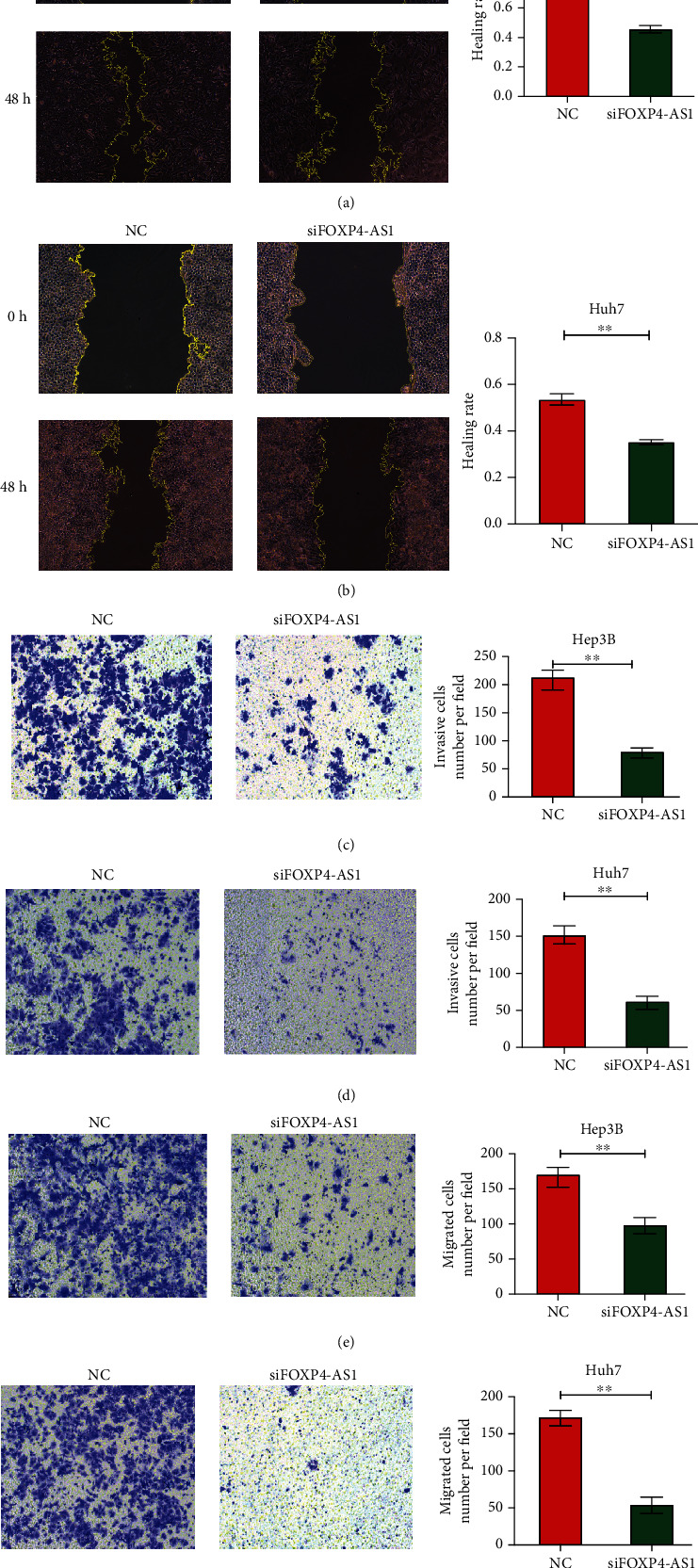
Effects of FOXP4-AS1 on Hep3B and Huh7 cells migration and invasion in vitro. (a, b) Cell scratch assay was used to detect the effect of FOXP4-AS1 on cell migration. The effects on cell migration and invasion were determined with the cell transwell test. (c) Detection of Hep3B cell invasion. (d) Detection of Huh7 cell invasion. (e) Hep3B cell migration assay. (f) Huh7 cell migration assay. All data are representative of three independent experiments and presented as mean ± s.d.

Moreover, we investigated the effect of FOXP4-AS1 on the migration and invasion of hepatoma cells by scratch test and transwell analysis. The scratch experimental results revealed that the migration abilities of the Hep3B (*P* < 0.001, Figure 3(a)) and Huh7 (*P* < 0.01, Figure 3(b)) HCC lines decreased evidently after FOXP4-AS1 was knocked down. Transwell assay displayed that the migration and invasion ability of the siFOXP4-AS1 group were remarkably lower than those of the NC group (*P* < 0.01, Figures 3(c)–3(f)). These results manifested that the FOXP4-AS1 downregulation inhibited the proliferation, migration, and invasion of the HCC cells.

### 3.4. Knockdown of FOXP4-AS1 Inhibited Angiogenesis

**Figure 4:**
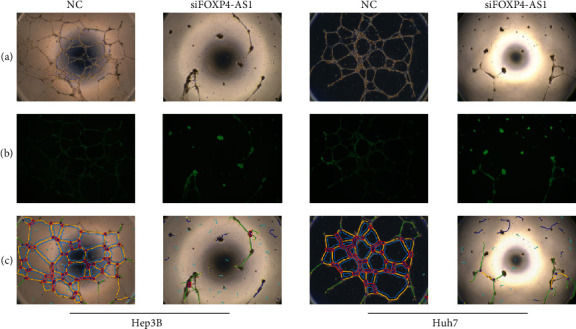
Detection of FOXP4-AS1 affecting neovascularisation ability. (a) Light microscope image. (b) Fluorescence image. (c) Computer postprocessing image.

HUVECs were cultured with different supernatants of transfected cells to validate the effect of FOXP4-AS1 on neovascularisation. It was observed that the angiogenesis abilities of the siFOXP4-AS1 group were obviously weaker than the NC group, and the number of vascular nodes, crossing points, mesh number, vascular branches, and trunk length differed significantly (*P* < 0.05, Figure 4).

## 4. Discussion

In the past decade, HCC has become one of the most common malignancies. Currently, the therapy for advanced HCC includes surgery, chemotherapy, and targeted drug therapy. However, the prognosis of patients with HCC remains pessimistic owing to the high recurrence rate and chemotherapy resistance [9]. Therefore, it is urgent to explore the mechanism of the HCC progression and explore new prognostic predictors.

lncRNAs are a class of noncoding RNA transcripts with >200 nucleotides [10]. As a pivotal regulator of certain genes and signal pathways related to tumourigenesis, mounting evidence indicates that lncRNAs are associated with numerous cell biological processes, incorporating cell proliferation, autophagy, apoptosis, invasion and metastasis, metabolism, and exosome secretion [11–15]. Emerging research has revealed that the deregulation of lncRNAs plays a part in the occurrence and development of HCC. Many lncRNAs such as LINC01419 [16], MFI2-AS1 [17], and LINC00160 [18] have been reported to be upregulated in HCC. Consistent with these lncRNAs, FOXP4-AS1 was greatly overexpressed in HCC tissues and cells in this study. Furthermore, lncRNAs are also used to predict the prognosis of multiple tumours. For example, lncRNA MNX1-AS1 and LINC00346 are upregulated in gastric cancer and predict poor prognosis [19, 20]; overexpression of lncDQ manifests low OS of patients with HCC [21]. Wu et al. [22] reported in their study that lncRNA FOXP4-AS1 is a prognostic indicator in prostate cancer. Actually, the worth of lncRNAs on evaluating the prognosis of patients with HCC could contribute guidance for postoperative adjuvant therapy. However, the clinical and potential biological functions of FOXP4-AS1in HCC have not been completely reported. Our results revealed that FOXP4-AS1 was highly expressed in HCC and was an independent risk factor for DFS and OS in patients with HCC, indicating that FOXP4-AS1 could be counted as a marker to predict the prognosis of HCC.

Furthermore, the abnormal expression of lncRNAs is closely related to tumour differentiation, proliferation, and metastasis [23]. Accumulating studies indicate that lncRNAs play a vital role in many biological and behavioural processes (cell proliferation, migration, invasion, and angiogenesis) of HCC [24–26]. For example, Zhan et al. [27] found that HOXA11-AS can promote the proliferation and invasion of HCC and induce epithelial-mesenchymal transformation. Besides, Kong et al. [28] confirmed in their research that silencing OTUD6B-AS1 obviously reduced the proliferation and invasion of HCC cells. In contrast, the overexpression of otud6b-as1 produced the contrary results. In addition, the study by Wang et al. provided proofs that angiogenesis in HCC is impeded by the silencing of BZRAP1-AS1. Our experimental results demonstrate that FOXP4-AS1 can significantly increase the cloning and proliferation abilities of HCC cells. The siFOXP4-AS1 group showed significant decreases in invasion, migration, and angiogenesis compared with the NC group. Therefore, in the future, this in-depth study of FOXP4-AS1 may be used to improve the high recurrence rate and high metastasis rate of patients with HCC. The cell function experiment fully confirmed the biological effect of FOXP4-AS1 on the growth and metastasis of liver cancer cells. Future studies will continue to evaluate the specific mechanism and pathway of FOXP4-AS1. As previously mentioned, FOXP4-AS1 is a FOXP4 antisense RNA; past studies have also pointed out that it will induce the expression of FOXP4. For example, it has been reported that lncRNA FOXP4-AS1 promotes the growth of prostate cancer by sequestering miR-3184-5p to upregulate FOXP4 [22]. In addition, FOXP4-AS1 positively regulated FOXP4 by interacting with insulin-like growth factor 2 mRNA-binding protein 2 (IGF2BP2) to stabilize FOXP4 mRNA in esophageal squamous cell carcinoma [29]. Interestingly, Zhang and Zhang discovered that FOXP4 was highly expressed in HCC and could promote (epithelial-mesenchymal transition) EMT of HCC cell line by regulating slug [30]. However, the role and molecular mechanism of FOXP4-AS1 and FOXP4 in hepatocellular carcinoma are still unclear. Our study confirmed that FOXP4-AS1 is a prognostic factor and affects the biological function of HCC. Further studies are needed to verify the specific mechanism of FOXP4-AS1 regulation and the correlation between FOXP4-AS1 and FOXP4 in HCC. In addition, our results need to be further verified in vivo. We will continue to explore in future experiments.

## 5. Conclusion

In summary, our study showed that FOXP4-AS1 overexpression in HCC is closely related to the prognosis of HCC. Moreover, the downregulation of FOXP4-AS1 inhibited the proliferation, invasion, and migration capacities of HCC, suggesting that FOXP4-AS1 plays a role as an oncogene in the occurrence and development of HCC cells, which provides a novel orientation for finding new therapeutic targets and potential prognostic biomarkers of HCC.

## Figures and Tables

**Table 1:** Clinicopathological features by FOXP4-AS1 expression levels.

	Training set				Validation set				Combined set			
Clinicopathologic parameters	Total (*n* = 87)	Low (*n* = 34)	High (*n* = 53)	*P* value	Total (*n* = 34)	Low (*n* = 11)	High (*n* = 23)	*P* value	Total (*n* = 121)	Low (*n* = 45)	High (*n* = 76)	*P* value
Gender				0.505				0.239				0.978
Female	13	4	9		3	2	1		16	6	10	
Male	74	30	44		31	9	22		105	39	66	
Age (years)				0.001				0.580				0.001
<60	69	21	48		30	9	21		99	30	69	
≥60	18	13	5		4	2	2		22	15	7	
Family history				0.142				0.580				0.532
No	79	33	46		30	9	21		109	42	67	
Yes	8	1	7		4	2	2		12	3	9	
AFP				0.002				0.138				<0.001
<400 ng/ml	43	24	19		12	6	6		55	30	25	
≥400 ng/ml	44	10	34		22	5	17		66	15	51	
HBVDNA				0.478				1.000				0.422
Negative	22	10	12		8	3	5		30	13	17	
Positive	65	24	41		26	8	18		91	32	59	
Metastasis				0.142				1.000				0.367
No	79	33	46		29	9	20		108	42	66	
Yes	8	1	7		5	2	3		13	3	10	
Tumor diameter				<0.001				0.070				<0.001
<5 cm	18	14	4		6	4	2		24	18	6	
≥5 cm	69	20	49		28	7	21		97	27	70	

AFP: alpha-fetoprotein; HBVDNA: hepatitis B virus-dexoyribonucleic acid.

**Table 2:** Cox regression analyses of factors predicting disease-free survival and overall survival of HCC.

Characteristic	Training set			Validation set			Combined set		
	HR	95% CI	*P* value	HR	95% CI	*P* value	HR	95% CI	*P* value
DFS									
Gender	1.174	0.506-2.727	0.708	1.024	0.446-2.355	0.955	2.447	0.874-6.849	0.088
Age	1.022	0.406-2.576	0.963	1.642	0.310-8.701	0.560	0.521	0.193-1.401	0.196
Family history	1.687	0.681-4.181	0.259	1.510	0.273-8.371	0.637	0.733	0.305-1.761	0.487
AFP	1.691	0.876-3.266	0.118	0.425	0.104-1.740	0.234	1.632	0.865-3.080	0.131
HBVDNA	2.353	1.038-5.337	0.041	0.546	0.135-2.204	0.395	2.010	1.019-3.965	0.044
Metastasis	1.226	0.482-3.116	0.668	2.370	0.502-11.177	0.276	1.129	0.519-2.456	0.760
Tumor diameter	1.515	0.557-4.117	0.416	4.458	0.466-42.604	0.194	0.956	0.421-2.171	0.914
FOXP4-AS1 expression	2.574	1.126-5.885	0.025	6.826	1.228-37.945	0.028	2.548	1.249-5.195	0.010
OS									
Gender	1.208	0.522-2.796	0.659	1.163	0.498-2.718	0.727	1.384	0.617-3.101	0.430
Age	1.036	0.408-2.626	0.941	1.689	0.327-8.732	0.532	0.643	0.215-1.919	0.849
Family history	1.399	0.566-3.459	0.468	2.248	0.406-12.454	0.354	1.474	0.683-3.184	0.323
AFP	1.727	0.888-3.361	0.107	0.312	0.077-1.259	0.102	1.317	0.740-2.344	0.349
HBVDNA	3.086	1.366-7.127	0.008	0.384	0.091-1.622	0.193	1.676	0.864-3.250	0.127
Metastasis	2.003	0.772-5.198	0.153	2.829	0.602-13.285	0.188	1.309	0.612-2.801	0.487
Tumor diameter	1.204	0.418-3.465	0.730	6.600	0.633-68.799	0.115	1.667	0.674-4.120	0.269
FOXP4-AS1 expression	2.712	1.140-6.450	0.024	6.505	1.165-36.399	0.033	3.012	1.441-6.297	0.003

AFP: alpha-fetoprotein; HBVDNA: hepatitis B virus-dexoyribonucleic acid.

## Data Availability

The data used to support the findings of this study is available from the corresponding author upon request.
